# From adhesion to biofilms formation and resilience: Exploring the impact of silver nanoparticles-based biomaterials on *Pseudomonas aeruginosa*

**DOI:** 10.1016/j.bioflm.2025.100267

**Published:** 2025-02-27

**Authors:** Maya Rima, Christina Villeneuve-Faure, Ludovic Pilloux, Christine Roques, Fatima El Garah, Kremena Makasheva

**Affiliations:** a LGC, University of Toulouse, CNRS, INPT, Toulouse, France; b LAPLACE, University of Toulouse, CNRS, INPT, Toulouse, France

**Keywords:** Silver nanoparticle-based biomaterials, plasma (gas discharge) process, Biointerfaces, Antimicrobial, Antiadhesion, Antibiofilm, *Pseudomonas aeruginosa*

## Abstract

Colonization of medical devices by microorganisms, often progressing to the formation of resilient biofilms, presents a common clinical issue. To address this challenge, there is growing interest in developing novel biomaterials with antimicrobial/antibiofilm properties as a promising preventive measure. This study explores nanocomposite biomaterials based on silver nanoparticles (AgNPs) deposited on thin silica (SiO_2_) layers for their potential effect on the adhesion, detachment, viability and biofilm formation of the opportunistic *Pseudomonas aeruginosa*. The AgNPs-based biointerface effect on biofilm development is investigated on the PAO1-Tn*7*-*gfp* strain by combining experiments under static and dynamic conditions. For the latter, a shear-stress flow chamber is used to mimic conditions encountered around certain medical devices. The findings reveal a rapid bactericidal effect of the AgNPs, noticeable within 30 min of exposure. Moreover, a delay in surface colonization is observed with a thin and unstructured biofilm, even after 72h of dynamic culture. A considerable fragility and sensitivity to hydrodynamic stresses is noticed for this loosely attached bacterial monolayer when compared with the thick and resilient biofilm formed on SiO_2_ surface. This study underlines the potential of AgNPs-based biomaterials in the conception of novel antimicrobial/antibiofilm surfaces with controlled release of the biocidal agent.

## Highlights

• Consideration of all bacterial colonization phases is indispensable to define the cell interactions with novel biomaterials.
• AgNPs and wall shear-stress shows the synergetic effect of employing multiple stresses for biofilm eradication.
• Significant delay of surface colonization and unstructured bacterial layer when in contact with AgNPs-based biomaterials.
• *P. aeruginosa* PAO1-Tn*7-gfp* does not organize in a structured biofilm when in contact with AgNPs-based biomaterials.
• AgNPs-based biomaterials have strong potential in the conception of novel antimicrobial surfaces with controlled release.

## 1 Introduction

Although the deployment of medical devices is commonly recognized as life-saving intervention, in some cases, it can be associated with serious clinical damages, including severe infections. The risk of microbial contamination, medical device colonization, then infection exists for medical devices and remains non-negligible during surgical interventions for the implanted ones, developing in some cases the co-called Implant-Associated Infections (IAIs) [[1], [2], [3], [4], [5]]. Indeed, the healthcare equipment, whether implanted or not, has the potential to act as a reservoir for microorganisms and to support their growth and proliferation. Once contaminated, devices may lead to severe chronic infections, along with impairment of the host's defense system and tissue necrosis for implanted devices [6,7]. One of the main causes of medical devices-related infections is the formation of a complex resilient structure known as “biofilm” [8]. This microbial community is defined as an aggregation of microorganisms attached to biotic or abiotic surfaces and enclosed in a slimy extracellular polymeric matrix (EPS) [9]. The transition from planktonic to sessile life, and the formation of structured biofilms, occurs in different steps [10]. Briefly, after microbial adhesion to the surface, cells multiply and proliferate along with EPS matrix production leading to the formation of a mature biofilm. Then, to ensure biofilm's life cycle, a dispersion step proceeds. Released microbes can therefore colonize other sites, ensuring the propagation of biofilm-related infections. The severity of this microbial community lies in its ability to overcome harsh environmental conditions, tolerate high concentrations of antimicrobial agents, and escape from the host immune response in the case of clinical infections [11].

Nowadays, despite the tremendous efforts devoted to the search for effective solutions, the treatment of biofilms formed on medical devices, whether implanted or not, is difficult and sometimes even impossible. Indeed, the available options for implanted devices are antibiotherapy that often fails and/or device removal. For the non-implanted ones, cleaning, disinfection and sterilization are the currently applied practices, but in spite these efforts, the outbreaks remain a threat. Such approaches are costly, leading to impairment of quality of life and patient outcomes and large economical losses for the healthcare systems [4,12]. Prevention strategies are thus essential to overcome the difficulties related to the control of an already structured biofilm [13]. Among the existing practices, environmental and staff control have proven their efficacy [14,15] but in terms of medical device protection there is still a lot of work to be done. In order to address this medical challenge, the development of medical devices with antimicrobial surfaces as biointerfaces, is considered a promising approach. In this context, the physicochemical modifications of the surfaces in general, and the incorporation of biocidal metal inclusions under nanoparticle form in particular, have emerged as the most promising strategies [16].

Nanoparticles offer a distinct strength due to their physical properties, notably their nanoscale structuring, high surface-to-volume ratio and high reactivity. These features enhance interactions with the target entities, thereby alteration in the corresponding function, even at a microbial cell level [17]. Among the emerging antimicrobial and antibiofilm nanoparticles, such as gold (Au), zinc (Zn), and copper (Cu) ones, the silver nanoparticles (AgNPs) receive great attention due to their inherent antimicrobial properties [[18], [19], [20], [21], [22], [23], [24]]. In fact, several routes of antimicrobial action are assigned to the AgNPs, including interaction with the cell membrane, leading to alterations in its structure and thus functions. AgNPs are also able to penetrate into the host cell, initiating a wide range of damages that disrupt the cellular machinery [25].

A considerable multi-targeted antimicrobial effect can be attributed to ionic silver (Ag^+^) released after oxidation of the AgNPs upon exposure to air and moisture [26]. Indeed, the AgNPs are able to simultaneously affect multiple cell targets, thus being efficient against bacteria, fungi, as well as viruses [25,27]. More recently, the ability of AgNPs to inhibit biofilm formation was also considered [28,29]. However, the applied approach in these studies is mainly global, without distinction of the biofilm formation steps and often performed under static conditions [30,31]. Demonstration of the AgNPs antimicrobial and antibiofilm activity depends on the selected microbiological method but also on the AgNPs synthesis way. The AgNPs are often synthesized by chemical methods, using toxic chemicals, which may leave residual compounds and thus induce unpredictable side effects [32,33]. The majority of reported works focus on AgNPs dispersed in solution and gels, which in turn induces use limitations related to aggregation and fast oxidation [31,34].

**Fig. 1:**
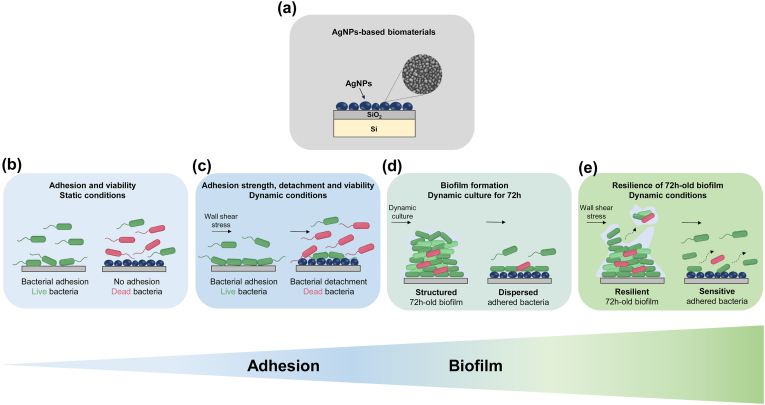
Schematic representation of the implemented strategy to evaluate the surface colonization phases by *P. aeruginosa* PAO1-Tn*7*-*gfp*, starting from adhesion to biofilm formation and resilience, when in contact with AgNPs: AgNPs-based biomaterials, composed of a single layer of AgNPs deposited on thin SiO_2_ layer thermally grown on Si-substrate (a), were assessed across the different stages of bacterial biofilm formation, under static and dynamic conditions. This holistic approach encompasses adhesion (b), adhesion strength (c), biofilm formation (d), and biofilm resilience (e).

To fill this gap and advance on the development of AgNPs-containing antimicrobial surfaces for potential integration in devices, our strategy, depicted in Fig. 1, relies on a physical synthesis method for elaboration of AgNPs-based biomaterials, namely a plasma-based process (electrical discharge), thus avoiding hazardous compounds and opening the way for compatibility with the current micro- and nano-technologies. Plasma-based deposition processes can be applied for coating the surfaces of substrates of different nature (among others metals, ceramics, polymers, natural substrates like horns, bones …), size, shape, morphology, etc. The deposit (dielectric, metallic or nanocomposite) is conformal to the substrate surface and can be applied to 3D-objects *e.g.*, implants. The plasma-based processes ensure homogenous, regular, and reproducible deposits on large surfaces. Moreover, they are considered safe compared to wet chemistry deposition methods and are at the origin of the current microelectronic Complementary Metal Oxide Semiconductor (CMOS)-technologies, which makes them advantageous for development of new devices. Thus, the selected plasma-based synthesis method for elaboration of AgNPs-based biomaterials involves free of ligands AgNPs, organized in a monolayer on the surface [[35], [36]]. It also ensures a homogeneous distribution of AgNPs in the plane [37,38], an important parameter to ensure regularity and reproducibility in the response of the microbial system. The structures studied in this work contain a monolayer of AgNPs immobilized on the surface of a thin SiO_2_ layer, thermally grown on Si-substrates (Fig. 1a). The choice of SiO_2_/Si substrates is based on the well-established biocompatibility and classification of SiO_2_ by the US Food and Drug Administration (FDA) as “Generally Recognized as Safe” (GRAS), making SiO_2_ suitable for medical applications. The SiO_2_ thin layers are also well-known for their optically transparent properties in the visible range of the spectrum and can successfully be used as antireflective coatings *e.g.*, glasses, contact lenses, *etc*. If properly dimensioned, the SiO_2_ thin layers can be used in plasmonics as a host matrix for biological studies [37,39,40]. Many nanotechnology devices for biomedical applications involve SiO_2_ thin layers, in particular when biosensing and/or CMOS-compatible bioelectronic devices are forecast [41]. By combining different characterization methods, the antibacterial and antibiofilm effects of these plasma elaborated AgNPs-based biomaterials are then assessed against *Pseudomonas aeruginosa*, a major nosocomial pathogen widely associated with medical devices-related infections [42]. The selection of *P. aeruginosa* is also supported by its natural resilience to antibiotics. Moreover, according to the very recent reports, *P. aeruginosa* emerges as highly prevalent pathogen in IAIs [5]. The particular choice of *P. aeruginosa* PAO1-Tn*7*-*gfp* is based on previous studies on this strain [43]. The originality of the developed strategy in this work resides in its holistic approach. By covering the entire chain of interactions *i.e.*, the different bacterial surface colonization phases, from microbial adhesion to biofilms formation and resilience, the impact of the synthesized AgNPs-based biomaterials on *P. aeruginosa* PAO1-Tn*7*-*gfp* is assessed. The study begins with evaluation of the microbial adhesion and viability under static conditions after different times of contact to assess the potential antiadhesive and bactericidal effects of the AgNPs-based biomaterials (Fig. 1b). Subsequently, the cell adhesion strength is determined under dynamic conditions, using a shear-stress flow chamber, to gain insight into the interaction between *P. aeruginosa* PAO1-Tn*7*-*gfp* and the AgNPs (Fig. 1c). The next explored phase is biofilm formation and cell proliferation under dynamic culture, targeting investigation of the potential antibiofilm effect of the AgNPs-based biomaterials (Fig. 1d). Lastly, the biofilm resilience under dynamic conditions is explored with combined hydrodynamic stress to unveil the interaction between the formed bacterial biofilm and the tested AgNPs-based biomaterials (Fig. 1e). Such strategy is of great interest to validate innovative approaches in the prevention of medical device associated infections since it simulates conditions that might be encountered around certain medical devices [44].

## 2 Experimental section

### 2.1 Elaboration of thin thermal silica (SiO_2_) layers

The thin SiO_2_ layers (80 nm-thick) used in this study were thermally grown on pre-cleaned Si-wafers (Sil'tronix) at 1100 °C under a controlled slightly oxidizing atmosphere (N_2_ with 1 % O_2_). The samples were pre-cut to always present an exposed surface of 1 cm^2^. The dimensions of the coupons were selected to fit into the shear-stress flow chamber.

### 2.2 Elaboration of AgNPs-based biomaterials by a plasma (electrical discharge) process

Previously prepared SiO_2_/Si samples were employed in the preparation of AgNPs-based biomaterials. The AgNPs deposition was performed in a hybrid plasma (electrical discharge) process successfully combining in the same reactor Ag-sputtering (Physical Vapor Deposition - PVD) and plasma polymerization (Plasma Enhanced Chemical Vapor Deposition - PECVD). This deposition method allows a strict control over the size and density of the AgNPs. Details about the plasma process and the methodology applied for deposition of the AgNPs-based biomaterials are provided elsewhere [37,38,45]. Briefly, an axially-asymmetric capacitively-coupled discharge was generated in the RF range, *f* = 13.56 MHz, and maintained in argon (AirLiquid AlphaGaz 2–99.9995 %) at low gas pressure (*p*_Ar_ = 5.4 Pa) with injected power of P = 80 W, that creates a self-bias voltage of V_dc_ = - 1000 V on the electrode covered by a silver target (Inland Europe, with purity of 99.99 %). The monolayer of AgNPs was deposited on the surface of the SiO_2_ thin layer by silver sputtering for 5 s. Before structural characterization and microbiological assays, the AgNPs-based biomaterials were stored under secondary vacuum (*p* = 0.06 Pa) in order to prevent from contamination and oxidation.

### 2.3 AgNPs-based biomaterials structural characterization

The thickness of the SiO_2_ thin layers was measured by spectroscopic ellipsometry with a SE-2000 ellipsometer in the 250–850 nm spectral range with an incident angle of 75°. Effective Medium Approximation (Bruggeman model) was applied to extract the thickness of the silica layers.

The AgNPs monolayer was studied with a Field Emission Gun (FEG)-Scanning Electron Microscope (SEM) JEOL JSM 7800F Prime. Images, recorded from the SEM observations were analyzed after their post-treatment for brightness and contrast.

Atomic Force Microscopy (AFM) topography analysis of the AgNPs-based biomaterials was performed in tapping mode with a Bruker Multimode 8 set-up using a TESPA-V2 probe.

In addition, Inductively Coupled Plasma – Optical Emission Spectroscopy (ICP-OES) analyses were conducted with an ICP-OES Horiba ULTIMA 2 spectrometer to quantify the released Ag^+^ ions from the AgNPs-based biomaterials after immersion in Water for Injectable Preparations (WIP, French Pharmaceutical Cooperation, Cooper, Melun, France) for 30 and 90 min. The selected times for Ag^+^ release measurements are representative of a step common to all different bacterial colonization phases explored and always performed under static conditions. Since the ICP-OES is a diagnostic method adapted for measurements under static conditions, only times up to 90 min are relevant for measuring the Ag^+^ release in this study. Briefly, a sample of 1.5 mL of solution containing the released Ag^+^ was introduced in the ICP argon plasma. The Ag spectral lines at λ = 328.068 nm and λ = 338.289 nm were followed. Since their intensity is considered proportional to the number of Ag species in the plasma, the quantity of released Ag^+^ ions was determined accordingly. The reported results are averaged over three independent measurements.

### 2.4 Bacterial strain and culture media

The bacterial strain used in this study was the opportunistic bacterium *Pseudomonas aeruginosa* (PAO1-Tn*7-gfp*, Tn*7* chromosomal insertion of *gfp*, constitutive expression of *gfp*, provided from the Department of Microbiology, University of Washington, Seattle, Washington, USA) [46] preserved at −80 °C in a cryoprotective solution.

Prior to each experiment, a first subculture was performed on Tryptic soy agar (TSA, Sigma-Aldrish, St. Quentin Fallavier, France) at 37 °C for 24h. The inoculum used in each experiment came from an overnight second subculture in Tryptic soy broth (TSB). To eliminate bacterial aggregates, the bacterial culture was filtered using a syringe filter (Cellulose Nitrate Syringe Filter, 5.0 μm, Cytiva Whatman) followed by centrifugation at 2400 rpm for 10 min at room temperature. The pellet was then rinsed in WIP in order to remove all the residual culture medium. The bacterial suspension was prepared in WIP and adjusted to an optical density of OD_640nm_ = 0.150 at λ = 640 nm, corresponding to a concentration of 10^8^ CFU/mL (CFU stands for Colony-Forming Unit). In all assays, the used final concentration of *P. aeruginosa* PAO1-Tn*7-gfp* suspension was 10^7^ CFU/mL also prepared in WIP in order to avoid possible interactions.

Concerning biofilm formation assay, a low nutritive medium, named minimum biofilm broth (MBB) was used, in order to create stressful conditions and subsequently promote biofilm formation and growth of adherent bacteria rather than planktonic growth [47,48]. The MBB (10X) medium is composed of FeSO_4_, 7H_2_O (0.005 g/L), Na_2_HPO_4_ (12.5 g/L), KH_2_PO_4_ (5.0 g/L), (NH_4_)_2_SO_4_ (1.0 g/L), glucose (0.5 g/L) and MgSO_4_, 7H_2_O (2.0 g/L) [49]. All these components were purchased from Sigma–Aldrich, St. Quentin Fallavier, France.

### 2.5 Adhesion and viability of *P. aeruginosa* PAO1-Tn*7-gfp* in contact with AgNPs-based biomaterials under static conditions

The adhesion and viability of *P. aeruginosa* PAO1-Tn*7*-*gfp* in contact with the AgNPs-based biomaterials and SiO_2_-samples were evaluated under static conditions by placing the tested samples in a 12-well plate (Falcon, TC-treated, polystyrene). Wells were then filled with 1.5 mL of bacterial suspension (10^7^ CFU/mL, which corresponds to 1.5 × 10^7^ CFU/cm^2^ = 7.2 log CFU/cm^2^) and incubated for a contact time of either 30 or 90 min at room temperature. Given that contamination of medical devices may occur at different stages in the life of medical devices, even before implantation, the selection of room temperature is considered representative. Additional interest of performing this study at room temperature is to provide a baseline understanding of the cell/surface interaction.

After the corresponding contact time, both non-adhered (planktonic cells) and adhered bacteria were enumerated. For the latter, samples were rinsed twice in a 2.0 mL WIP bath to remove sedimented and/or weakly adhered bacteria, thus enabling the quantification of the initially adhered bacteria. Subsequently, they were scraped (for 1 min) in 1.0 mL of WIP with a sterile spatula to recover attached bacteria. The counts of both planktonic cells and recovered adhered cells were carried out after serial dilutions (10^−1^ to 10^−6^) in WIP, followed by inoculation of 900 μL onto TSA by inclusion. The number of CFU was counted after 48h of incubation at 37 °C and subjected to logarithmic transformation based on formula (1):

(1) $$\log\;of\;bacteria\;\left(\log\;CFU/cm^{2}\right)=\log\frac{number\;of\;colonies\;\left(CFU\right)}{Dilution\;factor\times inoculated\;volume\times surface}\;\text{.}$$

It should be noted that only live and cultivable bacteria were detected and thus quantified in this experiment. In accordance with the applied methodology for counting the CFU all values were expressed in log CFU/cm^2^, which in this case corresponds and is equal to log CFU/mL.

On the other hand, for confirmation of bacterial surface colonization and to differentiate between live and dead adhered bacteria, some of the AgNPs-based biomaterials, as well as the identically processed SiO_2_-samples, were dedicated to epifluorescence microscopic observation after addition of iodide propidium (PI) (1 mg/mL prepared in WIP, PI final concentration = 0.1 μg/mL; Sigma-Aldrich, St. Quentin Fallavier, France). The observations were conducted using a Zeiss-Axiotech epifluorescence microscope with a 20X (Zeiss, EC Plan-Neofluar) objective, equipped with an HXP 120 C light source, a digital camera (Zeiss AxioCam ICm 1), coupled to the ZEN software.

### 2.6 Adhesion strength, detachment and viability of *P. aeruginosa* PAO1-Tn*7*-*gfp* in contact with AgNPs-based biomaterials under dynamic conditions

The adhesion strength was explored via evaluation of detachment profiles under hydrodynamic conditions [43,50]. The interaction with the AgNPs-based biomaterials and the viability of *P. aeruginosa* PAO1-Tn*7-gfp* were assessed and compared with results obtained for the bare SiO_2_-samples. To that end, a commercially available shear-stress flow chamber (10.13039/501100016409BST Model FC 71 Coupon Evaluation Flow Cell, BioSurface Technologies Corporation, USA) was used with a house-made customized coupon support, adapted to receive the tested samples and to ensure a uniform laminar flow. The experimental set-up is detailed in our previous publications [43,51]. To provide the possibility to apply a wide range of wall shear-stresses (0.01–5 Pa), it includes a syringe and peristaltic pumps, thus providing a broad spectrum of flow rates, ranging from very low to high, respectively. This is a very large step ahead to the simulation of wall shear-stresses encountered in physiological conditions [43,52] and especially to those exerted on implants in the human body [[53], [54], [55]], where blood flow can induce shear-stresses ranging from 0.1 Pa to 9.5 Pa. Very low values may also occur (0.005–1.5 Pa), especially at the ocular level.

Briefly, a *P. aeruginosa* PAO1-Tn*7-gfp* suspension of 10^7^ CFU/mL was initially injected into the shear-stress flow chamber containing the tested sample. This bacterial suspension was supplemented with 1.0 μL of PI (final concentration = 0.1 μg/mL) in order to differentiate between live (green) and damaged or dead (red) cells and thus to assess the potential bactericidal effect of AgNPs-based biomaterials. After injection, bacteria were maintained under static conditions for 90 min at room temperature, allowing bacterial settle and adhesion, while their viability was monitored every 30 min.

After the 90 min step, the number of initially sedimented cells (N_0_) was determined by epifluorescence microscopic observations. This initially observed cell count comprises both sedimented and adhered cells. In order to evaluate the detachment profiles of *P. aeruginosa* PAO1-Tn*7-gfp* adhered on SiO_2_-samples as well as on AgNPs-based biomaterials, the wall shear-stresses were incremented every 3 min and remained applied for 3 min in the range 0.01–5 Pa. The total number of cells (live and dead) remaining adhered on the surface (N) was determined in the observation area after the application of each shear-stress (at the end of the 3min-time slot). For comparison purposes, the percentage of bacteria remaining adhered was then calculated using formula (2):

(2) $$Cells\;remaining\;adhered\;\left(\%\right)=\frac{N}{N_{0}}\times 100\;\text{.}$$

The detachment profiles, representing the percentage of cells remaining adhered on the surface are plotted as function of the wall shear-stress. The detailed protocol, along with the range of applied wall shear-stresses (τ_p_, Pa), is provided in our previous publication [43].

### 2.7 Biofilm formation and resilience of *P. aeruginosa* PAO1-Tn*7*-*gfp* on AgNPs-based biomaterials under dynamic conditions

#### 2.7.1 Dynamic biofilm culture for 72h

**Fig. 2:**
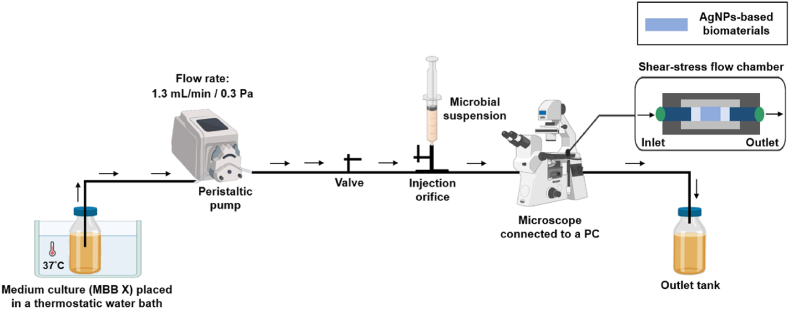
Schematic representation of the shear-stress flow chamber and the experimental set-up employed in the biofilm experiments.

The investigation of *P. aeruginosa* PAO1-Tn*7-gfp* biofilm formation and proliferation on the AgNPs-based biomaterials and the bare SiO_2_-samples, was conducted under dynamic conditions using the same shear-stress flow chamber as in the previous section, with some modifications in the experimental arrangement. A schematic representation of the modified experimental set-up is shown in Fig. 2.

After the 90 min contact time of the 10^7^ CFU/mL bacterial suspension prepared in WIP under static conditions at room temperature, a continuous flow of MBB (X) culture medium supplemented with PI (final concentration = 0.1 μg/mL) was applied for 72h and at a constant flow rate of 1.3 mL/min, corresponding to a wall shear-stress of 0.3 Pa. The constant wall shear-stress of 0.3 Pa was selected based on detachment experiments, demonstrating that for this value bacteria were not fully detached from the bare SiO_2_ layers and the AgNPs-based biomaterials. The 0.3 Pa wall shear-stress falls within the range of physiological stresses encountered in the body.

The growth of adhered bacteria in clinical conditions occurs *in vivo* and so, the culture medium reservoir was placed in a thermostatic water-bath at a constant temperature of 37 °C. Biofilm formation and proliferation on the various samples were recorded every 24h through epifluorescence microscopic observations (the same equipment as for the adhesion step), using an appropriate objective for observation at cellular level (2.7 pixel/μm) that is well-suited to our wall shear-stress flow chamber set-up. Fluorescence intensity (FI) of live and dead bacteria was determined using ImageJ (Fiji) software for three distinct images taken from the same experiment. This process was repeated for three independent experiments.

#### 2.7.2 Resilience of 72h-old biofilms

The resilience of biofilms formed on AgNPs-based biomaterials and on SiO_2_-samples after 72h of dynamic culture was assessed by using the shear-stress flow chamber. Briefly, following the 72h of biofilm dynamic culture, the wall shear-stress was increased two-fold every 3 min, and remained constantly applied for the 3-min time slot, ranging from 0.5 Pa to 16.7 Pa. Epifluorescence microscopic observations were carried out after the application of each shear-stress, followed by the determination of the FI of the remaining biofilms. FI values were then plotted logarithmically to facilitate the comparison between live and dead bacteria.

#### 2.7.3 Nanoscale imaging and analysis

The topography of the 72h-old biofilms formed on the AgNPs-based biomaterials and on the bare SiO_2_-samples was investigated by atomic force microscopy (AFM). The analyses were conducted with a Bruker Multimode mode 8 set-up in Tapping mode using a silicon-based tip (TESPA-V2). To prepare for measurements, samples were retrieved at the end of the biofilm experiments and thoroughly rinsed in 2.0 mL WIP bath. Subsequently, biofilms were fixed by immersing the samples in 1.5 mL of 4 % PFA (paraformaldehyde) for 20 min. Afterward, surfaces were rinsed twice in WIP and air-dried before observation. Measurements were performed in dry mode to avoid the mandatory step of microorganism's immobilization in liquid media. Indeed, the immobilization step requires a chemical treatment and/or a microstructuration of the surface which is incompatible with our goal to investigate the interaction of *P. aeruginosa* with the surface for bare SiO_2_-samples and AgNPs-based biomaterials.

### 2.8 Graphs and statistical analyses

SEM image processing of the AgNPs-based biomaterials and statistical analyses were performed with ImageJ 1.54d.

The graphs related to structural characterization of the AgNPs-based biomaterials and to AFM nanoscale analyses were generated with OriginPro 2022 9.9.0.220 (OriginLab Corporation).

The obtained results from microbial analyses were expressed as mean ± standard deviation (SD) from three independent experiments. The corresponding statistical tests, as well as the graphs were generated with GraphPad Prism 10.2.1 for Windows (GraphPad Software, USA). Statistically significant values were defined as a *p-*value (∗ < 0.05, ∗∗ < 0.01, ∗∗∗ < 0.001, ∗∗∗∗ < 0.0001).

## 3 Results

### 3.1 Structural characterization of AgNPs-based biomaterials

The AgNPs-containing structures employed in this work, called hereafter AgNPs-based biomaterials, were synthesized by sputtering of a silver target in the plasma of an axially-asymmetric electrical discharge maintained in argon at low gas pressure. All samples were of exposed surface of 1 cm^2^. Their structure comprised a single layer of AgNPs, deposited on the surface of thin SiO_2_-layers (80 nm thick), thermally grown on Si-substrates. The samples are highly reproducible, which allows for conduction of the delicate assays on bacterial adhesion and biofilm formation.

**Fig. 3:**
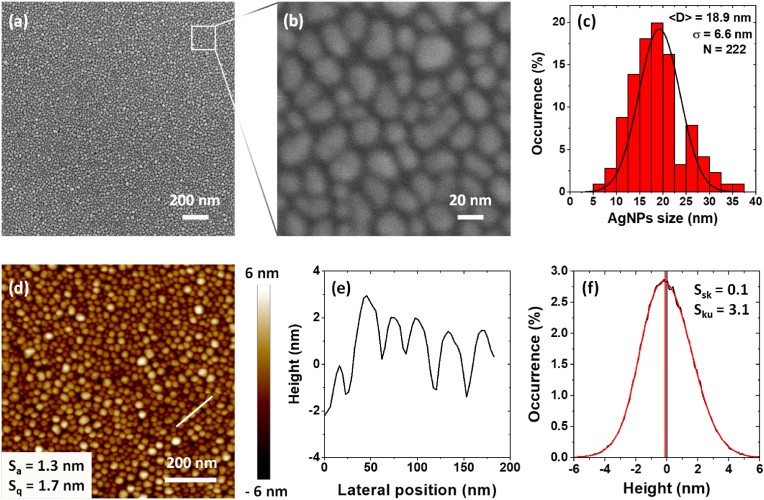
Structural characterization of the AgNPs-based biomaterials. (a, b) SEM top-view images at two resolution scales, (c) histogram showing results from statistics performed on 222 AgNPs: size distribution by bins and the corresponding Gaussian distribution function, (d) AFM topography image of the sample surface, including the arithmetic and quadratic surface height parameters, (e) AFM surface topography profile along 6 AgNPs, as marked with the white line on the topography image, and (f) extracted high order surface height parameters (skewness and kurtosis) of the studied area.

Fig. 3 shows the results from structural characterization of the AgNPs-based biomaterials performed by Scanning Electron Microscopy (SEM) and Atomic Force Microscopy (AFM). SEM images, taken at two different resolution scales (Fig. 3a and 3b), show top-views of the plasma synthesized AgNPs. The distribution of the AgNPs in the plane is regular and homogeneous, without free zones on the entire observation surface area of 4.0 μm^2^, as shown on the image (Fig. 3a). The same feature of the AgNPs-plane is observed all over the sample surface. High resolution SEM images show that the AgNPs-plane contains AgNPs isolated from each other (Fig. 3b). The AgNPs shape can be considered spherical, although a slight evolution towards a prolate spheroid, having eccentricity of less than 0.4, is observed. After SEM-image processing the AgNPs are found of average size of 18.9 ± 6.6 nm, as given on the size histogram in Fig. 3c. Their size distribution is well described by a Gaussian function. The AgNPs number surface density is found of 1.9 × 10^11^ NPs/cm^2^. This value originates from the 39.2 % SiO_2_ surface covered by AgNPs, as extracted from the SEM-images. Since the AgNPs are exposed, they convey a certain waviness to the sample surface, as shown in the AFM topography image (Fig. 3d) and profile (Fig. 3e). The surface topography analysis shows an arithmetic and a root mean square (quadratic) height parameter of S_a_ = 1.3 nm and S_q_ = 1.7 nm, respectively (Fig. 3d). Such low variance of the S_q_ height parameter, compared to S_a_, signifies that the AgNPs are organized as a single layer in the plane, as observed in the SEM images (Fig. 3a and Fig. 3b). The higher order height parameters (skewness and kurtosis) are found of S_sk_ = 0.1 and S_ku_ = 3.1, respectively (Fig. 3f). Although slightly positive, the very close to zero skewness value means that the topography of the AgNPs-plane is symmetrical about the center line *i.e.*, the AgNPs are with small dispersion in size (as found by the SEM observation) and randomly distributed on the sample surface, without free zones. The extracted kurtosis-value of 3.1, which is slightly larger but very close to the medium kurtosis level of 3.0 (Gaussian distribution of the spikes), confirms the normal distribution of AgNPs on the surface and points out to the spherical shape of these plasma synthesized AgNPs.

The targeted study of interaction between *P. aeruginosa* PAO1-Tn*7-gfp* and the AgNPs-based biomaterials requires quantification of the Ag^+^ ions released from the latter, since the reactivity and the stability of the samples are at play. The outcome of ICP-OES analysis after 30 and 90 min of immersion of the AgNPs-based biomaterials in WIP under static conditions shows a gradual release of Ag^+^ ions, with 75.0 ± 7.5 μg/L and 114.5 ± 6.9 μg/L being released after 30 min and 90 min of sample immersion, respectively. It means that the *P. aeruginosa* PAO1-Tn*7-gfp* species not only enter in direct contact with the AgNPs but also experience interactions with the Ag ^+^ ions they release.

### 3.2 Adhesion and viability of *P. aeruginosa* PAO1-Tn*7*-*gfp* in contact with AgNPs-based biomaterials under static conditions

**Fig. 4:**
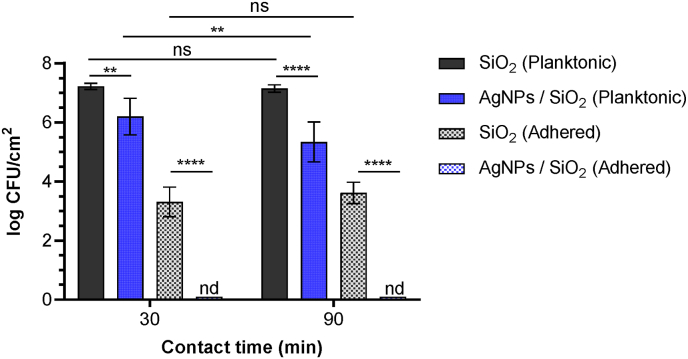
Study of the adhesion and viability of *P. aeruginosa* PAO1-Tn*7-gfp* on SiO_2_-samples and AgNPs-based biomaterials. Cultivable planktonic and adhered bacteria were quantified after 30 and 90 min of contact under static conditions. Results are expressed as means log CFU/cm^2^ ± Standard Deviation (SD) from three independent experiments. In accordance with the applied methodology for counting the planktonic cells log CFU/cm^2^ = log CFU/mL. Statistically significant differences are determined by two-way ANOVA with Tukey’ test for multiple comparisons (∗∗, *p-*value <0.01, ∗∗∗∗, *p-*value <0.0001), ns: not significant, nd: not detected. Epifluorescence microscopic images are presented in Supplementary Materials, Fig. S1.

The adhesion and viability of *P. aeruginosa* PAO1-Tn*7-gfp* on the AgNPs-based biomaterials were evaluated after 30 and 90 min of contact under static conditions. These results were compared to those on SiO_2_-samples used as controls in this study (Fig. 4). Assays included CFU-counts of adhered and planktonic cells and in the two cases differentiating between live and damaged/dead adherent cells.

Regarding planktonic bacteria, a significant reduction was observed after 30 min in presence of the AgNPs-based biomaterials (6.2 ± 0.6 log CFU/cm^2^) in comparison with the bare SiO_2_-samples (7.2 ± 0.1 log CFU/cm^2^). This reduction became even more pronounced after 90 min of interaction with the AgNPs-based biomaterials 5.3 ± 0.7 log CFU/cm^2^, compared to the remaining unchanged 7.2 ± 0.1 log CFU/cm^2^ population when in presence of the bare SiO_2_-samples. It should be noted that in this experiment, only cultivable viable bacteria were quantified, meaning that dead and damaged uncultivable cells were not detected.

Concerning adhered bacteria, the bare SiO_2_-samples were characterized by an adhesion level of 3.3 ± 0.5 log CFU/cm^2^ after 30 min of contact, representing a small part of the inoculum (of only 0.01 %). This value remained approximately the same after 90 min of contact with the bare SiO_2_-samples (3.6 ± 0.4 log CFU/cm^2^). Under the same conditions, cultivable adhered bacteria could not be detected on the AgNPs-based biomaterials after the 30 and the 90 min of contact time. Epifluorescence microscopic analysis conducted after 90 min of contact confirmed the absence of live and dead bacteria on the AgNPs-based biomaterials, while live bacteria were observed on the bare SiO_2_-samples (Supplementary Materials, Fig. S1).

These results demonstrate a rapid bactericidal effect of the AgNPs-based biomaterials on planktonic bacteria with a progressive impact in time. In the same time, they suggest the potential to limit the number of live adhered cells via direct bactericidal activity and/or anti-adhesive properties towards *P. aeruginosa* PAO1-Tn*7-gfp* attachment.

### 3.3 Adhesion strength, detachment and viability of *P. aeruginosa* PAO1-Tn*7*-*gfp* in contact with AgNPs-based biomaterials under dynamic conditions

The adhesion strength and the viability of *P. aeruginosa* PAO1-Tn*7-gfp* in contact with the AgNPs-based biomaterials were assessed under hydrodynamic conditions using a wall shear-stress chamber, after a sedimentation/adhesion step under static conditions in WIP. Prior to the assays, the bacterial viability during the sedimentation/adhesion step was determined on the SiO_2_-samples every 30 min, in the time slot of 0–120 min. These preliminary experiments were performed in the wall shear-stress chamber, as well. The number of live bacteria on the surface, within the area of observation, was considered stable after 90 min (Supplementary Materials, Fig. S2). Based on these results, the contact time for the sedimentation/adhesion step was defined to 90 min.

**Fig. 5:**
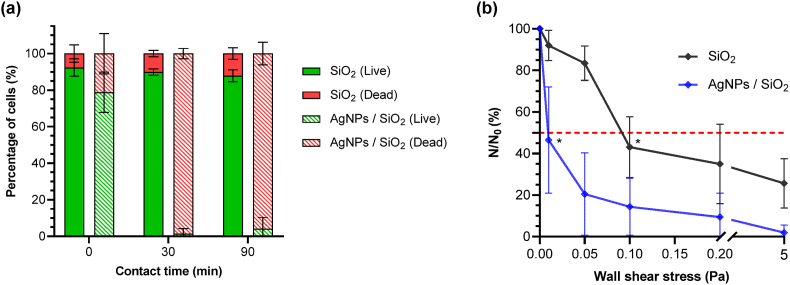
Percentage of live and damaged/dead *P. aeruginosa* PAO1-Tn*7-gfp* cells on SiO_2_-samples and AgNPs-based biomaterials at t_0_ (just after bacterial injection) and after 30 and 90 min of contact under static conditions in WIP in the shear-stress flow chamber (a). Wall shear-stress induced detachment profiles of *P. aeruginosa* PAO1-Tn*7-gfp* adhered on SiO_2_-samples and AgNPs-based biomaterials (b), (the red dash line on the figure shows the 50 % limit). Results are expressed as means ± SD from three independent experiments. Statistically significant differences determined by two-way ANOVA with Tukey's test for multiple comparisons (∗, *p*-value < 0.05) between t_90 min_ and after wall shear-stress application are indicated. (For interpretation of the references to color in this figure legend, the reader is referred to the Web version of this article.)

Concerning bacterial viability, the results show that the majority of *P. aeruginosa* PAO1-Tn*7-gfp* were alive upon injection (t_0_), regardless the tested surface (Fig. 5a). After 30 min of contact, 98.6 ± 2.8 % of bacteria were found damaged or dead on the AgNPs-based biomaterials, while the greater part of bacteria (89.9 ± 1.7 %) remained alive on the bare SiO_2_-samples. This bacterial viability on the SiO_2_-samples was maintained after the 90 min of contact.

The detachment profiles of *P. aeruginosa* PAO1-Tn*7-gfp*, recorded in the wall shear-stress experiment are presented in Fig. 5b. After the 90 min sedimentation/adhesion step under static conditions in WIP, the first and lowest wall shear-stress (0.01 Pa) was applied. In comparison with the initial number of bacteria (N_0_), 91.9 ± 7.2 % of cells remained adhered on the SiO_2_-samples. The 8.0 % loss of cells could be attributed to the removal of initially sedimented or weakly adhered bacteria [43]. A very distinct bacterial behavior was observed in contact with the AgNPs-based biomaterials. Significant detachment occurred upon the application of the first wall shear-stress (0.01 Pa), resulting in the release of approximately 50 % of the initially sedimented/adhered cells. It is worth to recall here that the majority of bacteria were found dead after the sedimentation/adhesion step of 90 min on the AgNPs-based biomaterials that preceded the dynamic evaluation (Fig. 5a).

Epifluorescence microscopic observations showed that the few bacteria remaining alive after the 90 min sedimentation/adhesion step on AgNPs-based biomaterials were removed upon the application of the first wall shear-stress (Supplementary Materials, Fig. S3). As a difference, observations conducted on the SiO_2_-samples showed conservation in the number and the viability of the cells. At larger applied wall shear-stresses, a gradual detachment of bacteria was observed on the AgNPs-based biomaterials, ending with a total cell detachment at the highest applied wall shear-stress (5.0 Pa). The controlled assays on bare SiO_2_-samples showed also a progressive decrease in the percentage of adhered cells, but with 25.7 ± 11.9 % of bacteria remaining adhered on the surface, even for the highest applied wall shear-stress.

### 3.4 Biofilm formation of *P. aeruginosa* PAO1-Tn*7*-*gfp* on AgNPs-based biomaterials under dynamic conditions

**Fig. 6:**
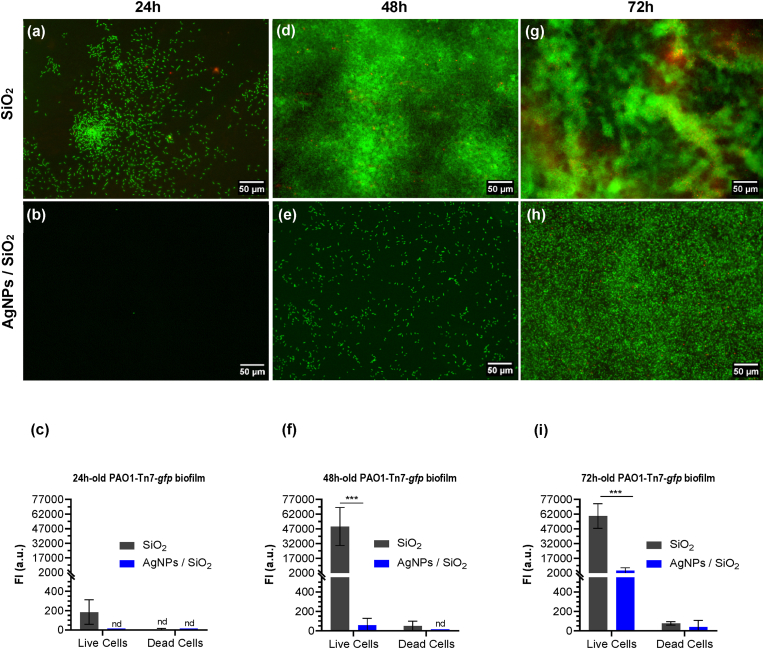
Biofilm formation by PAO1-Tn*7-gfp* with the corresponding fluorescence intensity (FI) (magnification x20, resolution 2.7 pixels/micron) on SiO_2_-samples and AgNPs-based biomaterials after 24 (a, b, c), 48 (d, e, f), and 72h (g, h, i) of dynamic culture*.* FI results for both live and damaged/dead bacteria are expressed as means ± SD from three independent experiments. Statistically significant differences determined by two-way ANOVA with Tukey’ test for multiple comparisons (∗∗∗, *p-*value <0.001) between SiO_2_-samples and AgNPs-based biomaterials are indicated. nd: not detected.

Biofilm formation and growth was evaluated under dynamic conditions using the same flow chamber and applying a constant flow rate of 1.3 mL/min, corresponding to a wall shear-stress of 0.3 Pa. Prior to the experiments, the 90 min sedimentation/adhesion step under static conditions in WIP was respected here as well. To promote the biofilm growth of *P. aeruginosa* PAO1-Tn*7-gfp*, rather than planktonic cell growth we used a low-nutritive medium (MBB). The MBB choice is based on previous experiments performed on *P. aeruginosa* [47,49]. The experiments were followed during 72h at 37 °C, with continuous supply of culture medium. Given the presence of multiple layers of cells, especially for advanced biofilms, the biomass was evaluated by FI measurements. For each of the three independent experiments, three observations were conducted on distinct zones. The results are presented as epifluorescence images of live and damaged/dead cells, at three steps of biofilm formation (24h, 48h and 72h) on AgNPs-based biomaterials and bare SiO_2_-samples (Fig. 6).

Within the first 24h of culture, a remarkable initiation of bacterial aggregate growth was observed on the bare SiO_2_-samples (Fig. 6a). Under the same conditions, no adhered bacteria were detected on the AgNPs-based biomaterials (Fig. 6b). It should be recalled here that within the detection limit of the applied characterization methods, no adhered cultivable bacteria were detected on the surface of the AgNPs-based biomaterials after the preliminary adhesion step under static conditions (90 min in WIP), combined with a significant reduction of planktonic cells (Fig. 4). These observations were supported by the 10.13039/100017146FI measurements (Fig. 6c).

Over time, relatively dense biofilms were observed on the SiO_2_-samples at 48h (Fig. 6d) whereas a small number of live adhered bacteria was detected on the AgNPs-based biomaterials (Fig. 6e). Despite the apparent lack of live adhered bacteria at 24h, the rare ones that could remain present were able to grow on the AgNPs-based biomaterials and became distinguishable at 48h. They organized on the surface under dispersed cells or small aggregates. The FI measured for the SiO_2_-samples was very high while it was almost inexistent for the AgNPs-based biomaterials (Fig. 6f). Indeed, the recorded FI for AgNPs-based biomaterials is reduced by 99.9 % in comparison to the bare SiO_2_-surfaces.

After 72h of dynamic culture, the experiment revealed the presence of a very few viable cells capable of multiplying in adhered form thus resulting in a slow and progressive colonization of the AgNPs-based biomaterial samples relative to the SiO_2_-ones. The significant difference between the two types of surfaces was confirmed. Thick and well-structured biofilms were established on the SiO_2_-samples (Fig. 6g), in contrast to the dispersed cells on the AgNPs-based biomaterials, without any sign of three-dimensional (3D)-organization (Fig. 6h). According to the FI measurements, most of the bacterial cells were alive with both types of samples (SiO_2_ and AgNPs-based biomaterials) (Fig. 6i). The FI measurements confirmed the significant difference in the observed 72h-old biofilms formed on SiO_2_-samples and on AgNPs-based biomaterials with FI reduction of 93 %. Even though the adhered biomass was significantly reduced on the AgNPs-based biomaterials compared to the SiO_2_-samples, a noticeable growth of sessile cells was observed in time, between 24h and 72h.

**Fig. 7:**
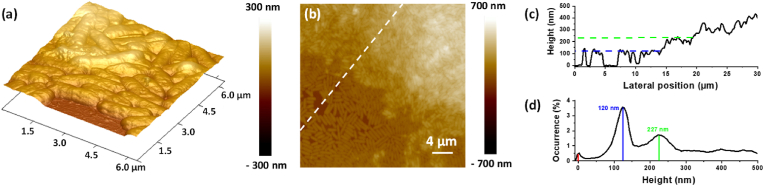
AFM topography images, in tapping mode, of 72h-old biofilm formed on SiO_2_-samples in 3D (6 μm × 6 μm) in (a) and 2D (32 μm × 32 μm) in (b) representations. The respective heights and occurrences are shown in (c) and (d). The displayed height profile in (c) was determined along the white dashed line shown in (b) The occurrence diagram in (d) was determined over the entire topography map in (b).

**Fig. 8:**
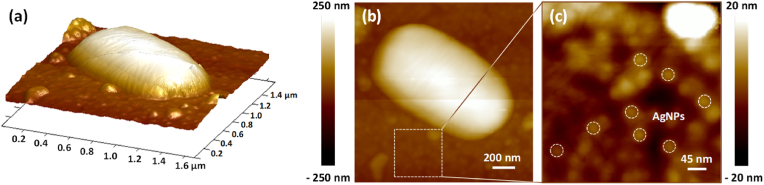
AFM topography images, in tapping mode, of AgNPs-based biomaterials after 72h of dynamic culture in 3D (1.6 μm × 1.6 μm) on (a) and 2D (1.6 μm × 1.6 μm) on (b) representations; the height color bar is common for the two representations, in (a) and (b). Magnification (425 nm × 425 nm) of the dashed square on (b), representing the remaining AgNPs (some of the AgNPs are suggested by the white dashed circles) is given on (c). (For interpretation of the references to color in this figure legend, the reader is referred to the Web version of this article.)

To complete the analyses, an AFM study in Tapping mode was conducted on dried samples, in order to investigate the topography of the 72h-old biofilms/adhered bacteria on the SiO_2_-samples and AgNPs-based biomaterials. The results for the two types of surfaces are summarized in Fig. 7, Fig. 8, respectively. Concerning the SiO_2_-samples, the topography shows a mass of bacteria covering the entire surface, in both 3D (Fig. 7a) and two-dimensional (2D) (Fig. 7b) representations. Fig. 7a focusses on the first step of biofilm formation. Indeed, this 3D-picture highlights the build-up of the bacterial first layer. The large biomass, observed on the SiO_2_-samples, allowed to track the evolution of the surface height profile, along the white dashed line (Fig. 7b). The resulting profiles are presented on Fig. 7c and 7d, in terms of height distribution (Fig. 7c) and occurrence (Fig. 7d). Fig. 7c confirms that the cells start their organization in a discontinues monolayer that gradually evolves to a multilayer structure, as well represented on the 2D-map (Fig. 7b). The extracted occurrence diagram (Fig. 7d), as determined over the entire topography map, reveals three distinguished peaks, positioned at 0 nm, 120 nm and 227 nm, and a continuum. As the measurements were performed in dry media, bacteria/biofilm heights are underestimated, whereas the measurement media has no impact on the shape and organization of bacterial population [56]. The 0 nm occurrence refers to the free zones (no bacteria on the surface). The highest occurrence, observed at 120 nm is correlated with the monolayer part of cell organization. The third peak (227 nm) appears with smaller occurrence and could be attributed to the progressive 3D-structuration in line with the following continuum. Moreover, this last part is characterized by a loss of contrast in the profile which demonstrates that the bacteria are embedded in a large EPS matrix.

After 72h of dynamic culture on the AgNPs-based biomaterials, the surface colonization is dispersed without any 3D-structuring (Fig. 6h). Such situation represented an interest to focus the AFM study on a single bacterium (Fig. 8). The 3D-representation on Fig. 8a suggests an undamaged cell. The 2D-representation on Fig. 8b, allows to analyze the AgNPs. Magnification of the zone in the dashed square (Fig. 8c) reveals the presence of AgNPs, some of which are in contact with the cell, suggesting that the AgNPs remain immobilized on the SiO_2_-surface even after a 3-day flow, without significant reduction in their size. This permits a long-time interaction between the bacterial cells and the AgNPs.

### 3.5 Resilience of *P. aeruginosa* PAO1-Tn*7*-*gfp* 72h-old biofilm formed on AgNPs-based biomaterials under dynamic conditions

**Fig. 9:**
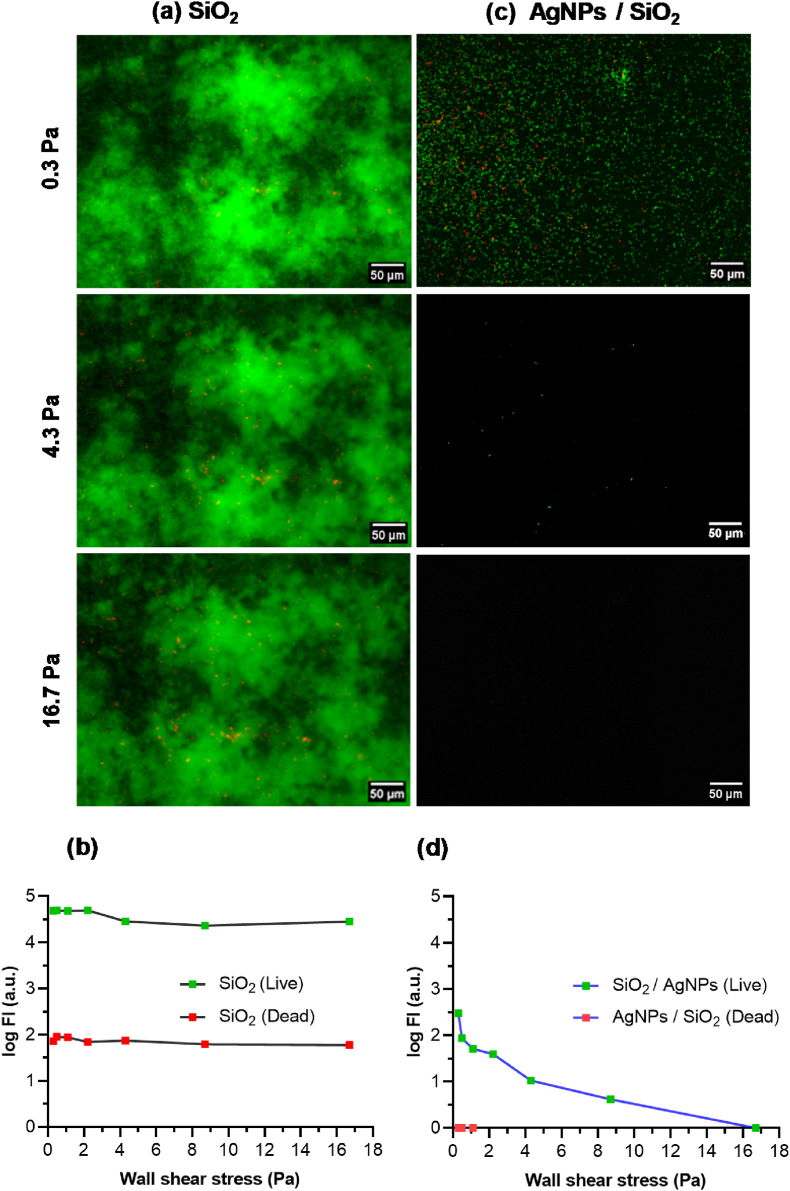
Epifluorescence microscopy images of *P. aeruginosa* PAO1-Tn*7- gfp* 72h-old biofilms formed on SiO_2_-samples (a), after application of low (0.3 Pa), medium (4.3 Pa) and very high (16.7 Pa) wall shear-stresses. Shear-flow induced detachment profiles of live and dead bacteria on SiO_2_-samples (b). The same sequence but for AgNPs-based biomaterials: epifluorescence microscopy images of *P. aeruginosa* PAO1-Tn*7- gfp* 72h-old biofilms (c) and wall shear-stress induced detachment profiles of live and dead bacteria (d).

Given the very different structures of 72h-old biofilms observed on the bare SiO_2_-samples and the AgNPs-based biomaterials, the detachment behavior of bacterial cells on the two types of surfaces was followed after application of increasing wall shear-stresses from 0.3 to 16.7 Pa. The results are expressed as log of the fluorescence intensity evolution for live and damaged/dead bacteria forming the 72h-old biofilms, within the same observation area (Fig. 9). Three typical values of the wall shear-stress were selected for the presentation of epifluorescence images, the lowest and the one constantly applied for the biofilm formation (0.3 Pa), one medium of 4.3 Pa, in relation with the results presented in Section 3.3, and a wall shear-stress of 16.7 Pa representing a very high flow rate (80 mL/min).

Fig. 9a presents the epifluorescence images recorded on the SiO_2_-samples. The 2D-biofilm morphology was preserved up to application of the highest wall shear-stress (16.7 Pa). Other observation zones presented the same behavior (Supplementary Materials, Fig. S4). The corresponding FIs were extracted for live and damaged/dead cells (Fig. 9b). The FIs of live cells confirm that the main part of adhered bacteria after the 72h-biofilm formation were still present and with the same organization (Fig. 6g) despite the variation of the wall shear-stress in the entire range, showing a very small evolution (within 0.2 log frame). The recorded FIs on damaged/dead cells present similar behavior (without strong variation), however with very reduced values. It is worth noticing that the cell viability was maintained even for high wall shear-stresses, up to the highest applied one. This clearly demonstrates the resilience of *P. aeruginosa* PAO1-Tn*7-gfp* 72h-old biofilms in response to all applied wall shear-stresses.

The behavior of the population adhered to the AgNPs-based biomaterials was markedly different (Fig. 9c). As noticed above, after the 72h biofilm formation, the bacteria were dispersed on the surface (Fig. 6h). A progressive detachment of residual sessile bacteria was observed for medium and very high wall shear-stresses, with no visible bacteria resisting the last condition. The recorded FIs of live bacteria (Fig. 9d) correlate with the epifluorescence images (Fig. 9c). The very low number of observed damaged/dead cells prevents the analysis of FI in the entire range of variation of the applied wall shear-stresses. It is worth noticing that as a difference from the condition for biofilm formation (0.3 Pa), the first increase of the wall shear-stress (from 0.3 to 0.5 Pa) led to a sudden reduction of the number of cells remaining adhered on the surface (Fig. 9d). Further increase of the wall shear-stress led to a progressive cell detachment. The highest applied wall shear-stress of 16.7 Pa entailed an apparent global detachment of the cells, with FI-value below 0.1 log. Altogether, these results show that in contact with AgNPs-based biomaterials, *P. aeruginosa* PAO1-Tn*7-gfp* does not organize in a structured biofilm, even after 72 h of culture. In addition, the flat bacterial layer, made up of dispersed cells on the surface of AgNPs-based biomaterials, appears very sensitive towards increasing wall shear-stresses, demonstrating no or poor resilience under dynamic conditions.

## 4 Discussion

Bacterial invasion of indwelling medical devices, frequently progressing to the development of resilient biofilms, is a prominent cause of device failure and reduced operational lifetime [6]. To limit the risk of biofilm formation, a lot of research was conducted in connection with development of new and better adapted materials for biomedical devices, in particular with antimicrobial properties [57,58]. Among others, nanotechnology-derived materials designed to coat these devices with an antimicrobial/antibiofilm nanosized layers able to prevent microbial colonization, represents a promising approach that is continuously progressing [59]. Silver nanoparticles are recognized as a non-specific antimicrobial agent able to simultaneously target multiple cellular processes and being effective on a broad spectrum of microbial species [[21], [22], [23], [24],[60], [61], [62]].

In the present study, we aimed to evaluate the potential antiadhesion and antibiofilm properties of the elaborated AgNPs-based biomaterials against the opportunistic bacterium *P. aeruginosa* [5]. The originality of the study lies in its holistic but yet comprehensive approach, covering all related aspects: elaboration and characterization of AgNPs-based biomaterials and evaluation of the different phases of biofilm formation and development, considering *P. aeruginosa* PAO1-Tn*7-gfp*.

The AgNPs-based biomaterials consisting of a monolayer of AgNPs deposited on a thin SiO_2_ layer grown on Si-substrate were plasma (electrical discharge) processed [36,38]. Indeed, anchoring of AgNPs on a surface offers increased stability compared to colloidal AgNPs, thereby reducing the risk of their aggregation and fast oxidation. It also provides high-level and long-term antimicrobial properties [32,33]. Regarding biofilm issues, Choi et al., have partially attributed the resistance of *Escherichia coli* biofilm against AgNPs in solution (ranging in size from 15 to 21 nm) to the AgNPs aggregation into larger particles, expanding up to 40-fold their initial size [63]. This aggregation can decrease interaction and diffusion of AgNPs within the biofilm, thereby reducing their antibiofilm efficacy. Such issues are avoided with AgNPs immobilized on the surface of SiO_2_/Si samples. There are however, some limitations for the AgNPs-based biomaterials discussed in this study. The main one is related to the fact that, although immobilized on the SiO_2_ surface the AgNPs can detach, since there are no covalent bonds between the surface Ag-atoms of the AgNPs and the SiO_2_-surface. Only weak forces are at play. The AgNPs may also experience different contamination processes due to their exposure, which may alter their biocidal efficacy. A way to overcome these issues is to embed the AgNPs in the SiO_2_-matrix which will allow to protect them but also to modulate the Ag^+^ release. In general, the biocidal activity of AgNPs is considered in terms of a dose/contact time pair. Embedding the AgNPs in the silica matrix will allow to modulate the released amount of Ag^+^ and/or AgNPs and to deliver the appropriate dose to the targeted microorganisms. Such strategy, that we follow in an ongoing project, will open the way of designing tailored antimicrobial surfaces with controlled biocidal activity and a reduced environmental impact.

The performed and presented here microbial study on *P. aeruginosa* PAO1-Tn*7-gfp* starts from adhesion and progresses through biofilm formation, addressing also the resilience of the formed biofilm. This integrated investigation was achieved by conducting experiments under both static and dynamic conditions in order to examine the adhesion, and under dynamic conditions only, by using a wall shear-stress flow chamber, to assess the impact on biofilm formation. Under static conditions, the proposed AgNPs-based biomaterials proved efficiency towards adhesion and viability of *P. aeruginosa* PAO1-Tn*7-gfp*. Assessment of the effect showed no detection of adhered cultivable bacteria on the surface after 30 and 90 min in WIP. At the same time, the number of non-adhered cells remaining in suspension, was significantly reduced after only 30 min (reduction of 1 log), in comparison to the control SiO_2_-samples for which no reduction was detected. A more pronounced bactericidal effect of the AgNPs-based biomaterials on planktonic cells was detected after 90 min in WIP (2 log reduction). Such behavior can be explained by combined antiadhesive and bactericidal activities (Fig. 4). Similar antibacterial activity of AgNPs, when partially embedded in an organosilicon matrix, was reported for *Escherichia coli* K12MG1655 after 4h of contact [64]. The bactericidal activity of AgNPs-based biomaterials is known to go through both a direct impact of the AgNPs and a progressive release of Ag^+^ ions [36,65,66]. Pugliara et al. demonstrated the toxicity of Ag ^+^ on algal photosynthesis after their release from AgNPs (size ≤20 nm) embedded in thin silica layers, close to the sample surface. In experiments on *Escherichia coli* MTCC 443, Agnihotri et al. demonstrated that direct contact was the predominant bactericidal mechanism of action of AgNPs-glass substrate compared to the released concentration of Ag^+^ [32]. To go further in the analyses made here, the release of Ag^+^ from AgNPs-based biomaterials was controlled by the ICP-OES measurements performed after 30 and 90 min of immersion in WIP under static conditions. The selected times for release are common to all different bacterial colonization phase exploration. The values of released Ag^+^ concentrations are in the range of bactericidal silver ion concentrations previously observed against Gram negative and Gram positive bacteria [67] and related to the combination of various mechanisms of action [[68], [69], [70]]. Considering the 1 cm^2^-surface of the AgNPs-based biomaterials under study, the released Ag-amount represents a very small fraction of the initial Ag-reservoir (evaluated to 2 % after 90 min). This value is in accordance with previously obtained results [36]. Therefore, the observed dual effect on adhered and planktonic bacteria can be attributed to the AgNPs antibacterial efficacy arising from both their direct contact impact, primarily dominant, and the subsequent gradual release of antibacterial Ag^+^ ions.

In order to explore the impact of our AgNPs-based biomaterials on *P. aeruginosa* PAO1-Tn*7-gfp* adhesion, further experiments were conducted under dynamic conditions. The latter allowed to analyze the adhesion strength of bacteria on the basis of detachment profiles. The employed flow rates (wall shear-stresses) were close to conditions encountered around some medical devices [52]. The viability of *P. aeruginosa* PAO1-Tn*7-gfp* was also followed. During the preliminary sedimentation step of 90 min of contact with the AgNPs-based biomaterials performed in the wall shear-stress chamber, a global mortality of sedimented/adhered bacteria was noted (Fig. 5a), in line with the previous experiment performed under static conditions (Fig. 4 and Supplementary Materials, Fig. S1). After the application of the lowest wall shear-stress (0.01 Pa), more than 50 % of sedimented/adhered cells were removed, with the majority being damaged/dead (Fig. 5b). This is a very important difference compared to the bare SiO_2_ surface, for which the majority of cells were viable and remained adhered. It is worth noticing that this very low wall shear-stress (0.01 Pa) can be considered comparable to the rinsing phase under the static condition experiment. Increase of the wall shear-stress conducted to a gradual removal of the bacterial population for the AgNPs-based biomaterials, with total removal attained at 5.0 Pa. A progressive reduction was also observed on the bare SiO_2_ surface, however keeping a positive difference of around 30 % of the bacterial population in the entire range, even for the highest applied wall shear-stress. An important conclusion can be made at this stage, namely the AgNPs-based biomaterials significantly impair the viability and the adhesion strengths of *P. aeruginosa* PAO1-Tn*7-gfp*, demonstrating a global impact on the adhesion step. It is worth noticing that the experiments here were performed without any added conditioning of the sample surface by proteins or other constituents of a natural surrounding environment. Depending on the proteins that condition the surface, their type, mixture of proteins, amount and their organization on the surface, the cell adhesion is altered [43,51]. The detachment strength, and consequently the detachment profiles under increasing wall shear-stresses is different, showing for example stronger microbial adhesion for SiO_2_-surfaces conditioned by fibronectin (Fn) layers compared to those conditioned by bovine serum albumin (BSA) ones. The antimicrobial efficacy of AgNPs is also modulated by the presence of proteins. Since the AgNPs are anchored on the surface, phenomena like AgNPs-uptake by the proteins, especially for Fn are likely. Such phenomena are worthy of exploring.

Considering the surface colonization phases, the initial microbial adhesion is followed by cell growth leading to biofilm formation. In order to assess the potential of AgNPs-based biomaterials to prevent or at least to disturb the biofilm formation, a continuous dynamic culture maintained at a low wall shear-stress (0.3 Pa) was conducted for 72h (Fig. 6). Based on previous studies, biofilms were grown in a minimal culture medium (MBB) *i.e.*, low-nutrient broth, as recommended by Yung et al., [71] in order to imitate the natural nutrient-limiting growth conditions and favour biofilm populations over planktonic bacteria [47,48]. Under our conditions discussed here, it means that the direct interaction of microorganisms with the surface is privileged. Moreover, it reveals the impact of biomaterials with immobilized AgNPs on their surface on sessile bacteria.

In direct contact of *P. aeruginosa* PAO1-Tn*7-gfp* with the AgNPs-based biomaterial samples, the experiment reveals very few viable cells capable of multiplying in adhered form thus resulting in a very slow and progressive surface colonization throughout the 72h of dynamic culture, relative to the bare SiO_2_-samples. Such behavior is not singular. It was previously revealed on other Gram-negative bacteria, however attributed to different inception mechanisms. Wirth et al.*,* demonstrated the ability of a small fraction of *Pseudomonas fluorescens*, having evaded the antibacterial effect of AgNPs-coated glass surface and of the released Ag^+^ ions, to recover and colonize the surface over time, thereby initiating biofilm formation [66]. The observed effect was inoculum dependent and was ascribed to a decrease in dissolved silver ion bioavailability. In the same vein, Allion-Maurer et al., evaluated the antibacterial and antibiofilm activity of plasma-deposited coatings containing AgNPs embedded in an organosilicon matrix and observed a decline in the silver's antimicrobial impact on *Escherichia coli* K12 MG1655 after 48h of static culture [64]. The authors attributed the initial efficiency to the coating bactericidal activity and to silver-mediated bacteriostatic effect. The gradual loss of activity after 2 days of contact was explained by an oxidation of both the organosilicon matrix and the AgNPs, with silver release at the extreme surface.

In our conditions, it is important to note that the AFM analysis of the AgNPs-based biomaterials after 72h of dynamic culture revealed continued presence of AgNPs on the surface (Fig. 8). High-resolution AFM analyses underlined no significant reduction of the AgNPs size. The stability of AgNPs is mainly related to oxidation processes, giving rise to the release of Ag^+^ ions. The oxidation is more important for smaller AgNPs (below 4 nm of size). In all cases the kinetic constants are long, especially at low temperature. The question of stability of AgNPs and the related oxidation processes is still under debate in the literature. Currently it is mainly treated numerically [72]. Experimental verifications require dedicated studies due to the very strong dependence on the environment [73]. Generally, the Ag^+^ release rate increases in the first hours and then stabilizes and remains stable over long time (up to 30 days) [[35], [74]]. The approximately 20 nm size of the AgNPs used in this study are expected not to suffer strongly the oxidation process and to stabilize the release rate. It is worth to recall here that in the end of the adhesion phase under static conditions (90 min), only 2 % of the total Ag amount was released, also demonstrating a slow and time-dependent silver release. Namely, this slight instability of the AgNPs allows for their effective interaction with bacteria. However, their long term antibacterial activity remains to be explored, especially considering also the permanent direct contact with cells. Due to the direct contact with the cells, our AgNPs-based biomaterials limit *P. aeruginosa* PAO1-Tn*7-gfp* surface colonization, resulting in a single layer of bacteria (Fig. 6e), greatly different from the mature, structured, dense, and thick biofilm developed on bare SiO_2_-samples (Fig. 6d). This can be considered as a simple colonization delay due to a bacteriostatic effect of silver or to a lack of bioavailable silver. Another hypothesis may be a deeper modification in the cell/surface interaction due to the direct contact with the AgNPs that can impact the biofilm resilience.

Generally speaking, the resilience of biofilms against antimicrobial agents and physical treatments is largely attributed to the dense bacterial population constituting the structured biofilm, as well as to the protective extracellular polymeric matrix surrounding it. In the case of AgNPs-based biomaterials, cells in direct contact with the AgNPs are rapidly damaged/killed and the few ones remaining alive are only able to organize under a bacterial monolayer, even after 72h of dynamic culture. This impacted population most likely presents a higher sensitivity toward other antimicrobial (physical or chemical) treatments, compared to the structured biofilm detected on the bare SiO_2_-samples. Such feature has been reported in the literature, in the case of silver-coated TiAlNb alloy cages, alone or associated to daptomycin or vancomycin that effectively prevented IAIs by *S. epidermidis in vivo* [3]. No emergence of silver resistance in several staphylococci strains was observed after 50 passages, thus concluding that silver coatings are a promising strategy for lowering the risk of IAIs. Another recent example of combined stress (ultrasound and antimicrobial polymeric nanoparticles) demonstrates additive to synergetic effect for bacterial biofilms eradication [75].

The tenacity and persistence of bacterial biofilms formed on medical devices to fluid flows and applied mechanical forces is a crucial feature in associated infections [76]. This characteristic reflects their ability to persist or detach and spread. Once the applied stress exceeds the biofilm's strength and cohesion, detachment and dissemination occur, increasing its sensitivity to antimicrobial agents and components of the host immune system [76,77]. In this respect, the resilience of the 72h-old biofilm grown on bare SiO_2_-samples is studied and compared with the unstructured one formed on the AgNPs-based biomaterials by applying increasing wall shear-stresses (physical treatment). While the structured biofilm, present on the SiO_2_-samples, remains predominantly intact even after applying the highest wall shear-stress (16.7 Pa), a progressive cell detachment is observed for the unstructured biofilm formed on the AgNPs-based biomaterials, ultimately leading to a complete detachment at 16.7 Pa (Fig. 9). These assays confirm the lack of or poor resilience of cells adhered on the AgNPs-based biomaterials. However, the fate of cells remaining adhered needs to be followed due to the risk of selection of resistant populations. These findings demonstrate that the AgNPs-based biomaterials have a decisive impact on the phases of adhesion and colonization under biofilm, leading to biofilm “fragility” and therefore sensitivity to *in vivo* defenses and associated treatments.

## 5 Conclusion

The holistic approach applied in the present study underlines the importance of considering the different bacterial surface colonization phases when the aim is to fairly describe the global picture of events and to link the information on cell interactions with novel biomaterial with antimicrobial/antibiofilm properties. By combining experiments under static and dynamic conditions, this study is the first to cover the microbial adhesion, the adhesion strength, the biofilm formation and the resilience of *P. aeruginosa* PAO1-Tn*7-gfp* in contact with AgNPs-based biomaterials. Moreover, by employing increasing wall shear-stresses to characterize cell/surface interactions it demonstrates the synergetic effect of multiple stresses for bacterial biofilms eradication.

The employed AgNPs-based biomaterials are fabricated by a plasma process (electrical discharge), thus avoiding the use of hazardous compounds and opening the way for compatibility with the current micro- and nano-technologies. Moreover, the plasma deposition process offers a wide range of applications, enabling the development of strategies for coating various types of clinically used implant materials. The AgNPs are immobilized on the surface of SiO_2_ thin layers, thermally grown on Si-substrates. Their distribution is regular and homogeneous, without free zones on the entire surface area. The AgNPs-based biomaterials are highly reproducible, which in turns ensures regularity and reproducibility in the response of the microbial system. The main finding of this study is that in contact with AgNPs-based biomaterials, *P. aeruginosa* PAO1-Tn*7-gfp* does not organize in a structured biofilm, even after 72 h of culture. In addition, the flat bacterial layer that forms on the surface of the AgNPs-based biomaterials is made up of dispersed cells and appears very sensitive towards application of increasing wall shear-stresses, thus demonstrating no or poor resilience under dynamic conditions. The release of bioavailable silver (Ag^+^ and/or AgNPs) is sustained in time and prevents from administration of a large quantity of ionic silver, thus avoiding human and environmental risks.

This original approach enables to demonstrate the possibility of offering AgNPs-based biomaterials with defined characteristics and thus to assess their specific antimicrobial properties in terms of bacterial adhesion, biofilm formation and ultimately resilience. This work opens the way to further explorations concerning the correlation between the size and the number surface density of AgNPs, the rate of Ag ^+^ release and the modification of adhesion and biofilm formation properties of *P. aeruginosa* PAO1-Tn*7-gfp*. Such strategy is of great interest to validate innovative approaches in the prevention of medical device associated infections since it allows to mimic the conditions encountered around certain medical devices. It paves the way for development of effective devices for prevention of implant-associated infections and thus can be considered for clinical application.

## CRediT authorship contribution statement

**Maya Rima:** Writing – original draft, Visualization, Investigation, Formal analysis, Data curation. **Christina Villeneuve-Faure:** Writing – review & editing, Visualization, Methodology, Investigation, Formal analysis, Data curation. **Ludovic Pilloux:** Writing – review & editing, Supervision. **Christine Roques:** Writing – review & editing, Validation, Supervision, Methodology, Formal analysis, Conceptualization. **Fatima El Garah:** Writing – review & editing, Supervision. **Kremena Makasheva:** Writing – review & editing, Project administration, Methodology, Investigation, Funding acquisition, Formal analysis, Data curation, Conceptualization.

## Declaration of competing interest

The authors declare the following financial interests/personal relationships which may be considered as potential competing interests: Kremena Makasheva reports financial support was provided by 10.13039/501100001665French National Research Agency. If there are other authors, they declare that they have no known competing financial interests or personal relationships that could have appeared to influence the work reported in this paper.

## Data Availability

Data will be made available on reasonable request.
