# Supplementary material for: From adhesion to biofilms formation and resilience: Exploring the impact of silver nanoparticles-based biomaterials on Pseudomonas aeruginosa
Source: Biofilm. 2025 Feb 27;9:100267. doi: 10.1016/j.bioflm.2025.100267 (PMC11930599; doi:10.1016/j.bioflm.2025.100267)
Supplement: Multimedia component 1 [file mmc1.docx]

**SUPPLEMENTARY MATERIALS**

**S1: Adhesion and viability of *P. aeruginosa* PAO1-Tn*7*-*gfp* in contact with AgNPs-based biomaterials under static conditions**


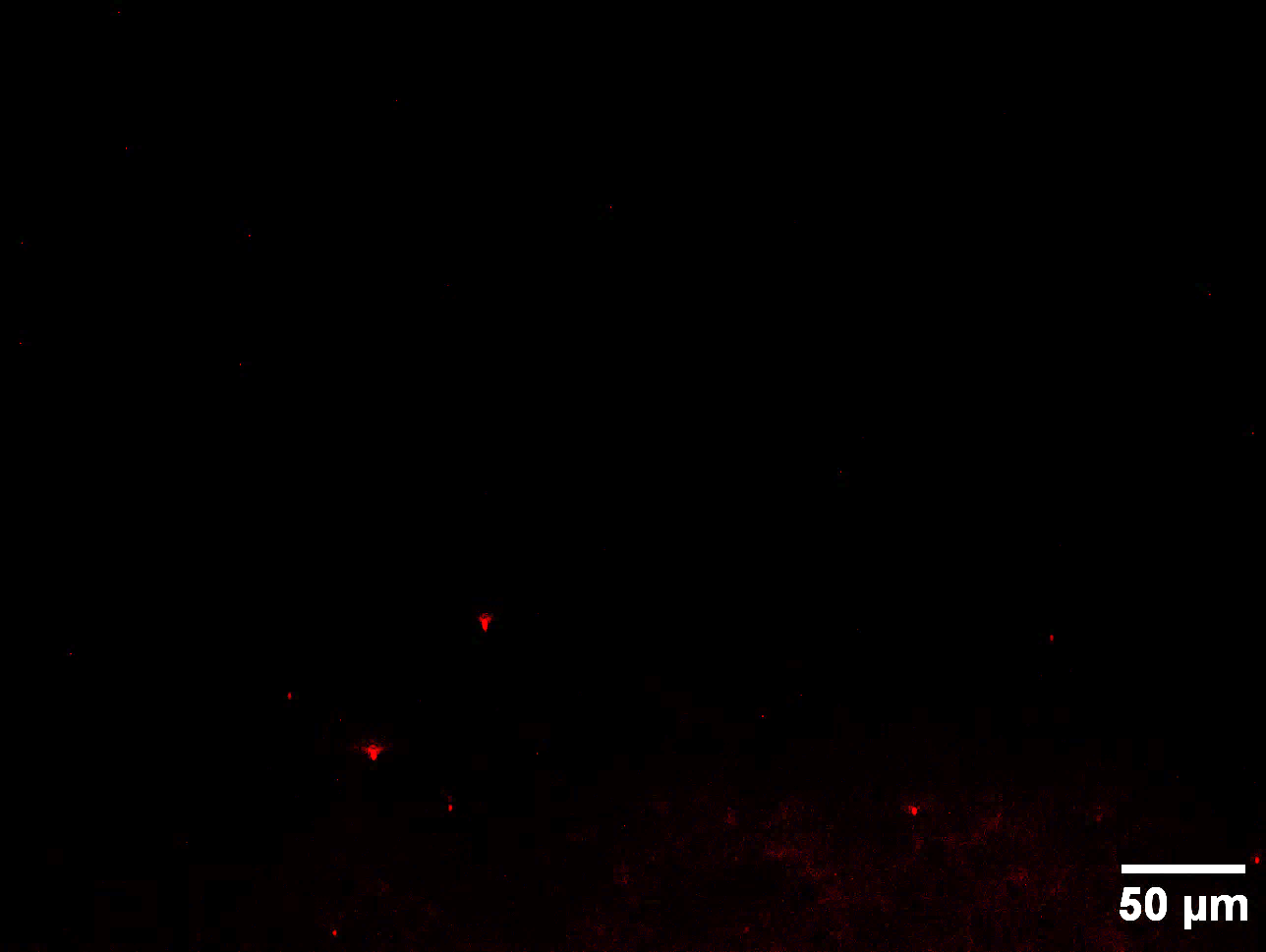

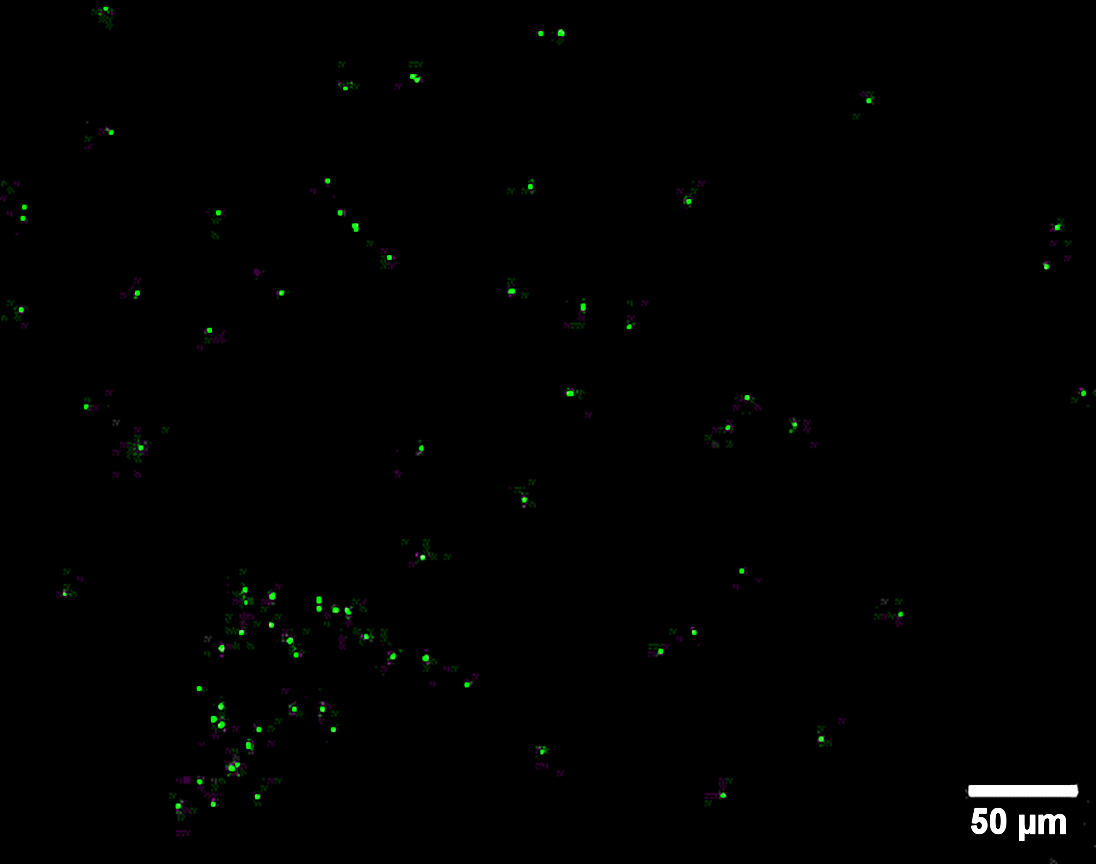

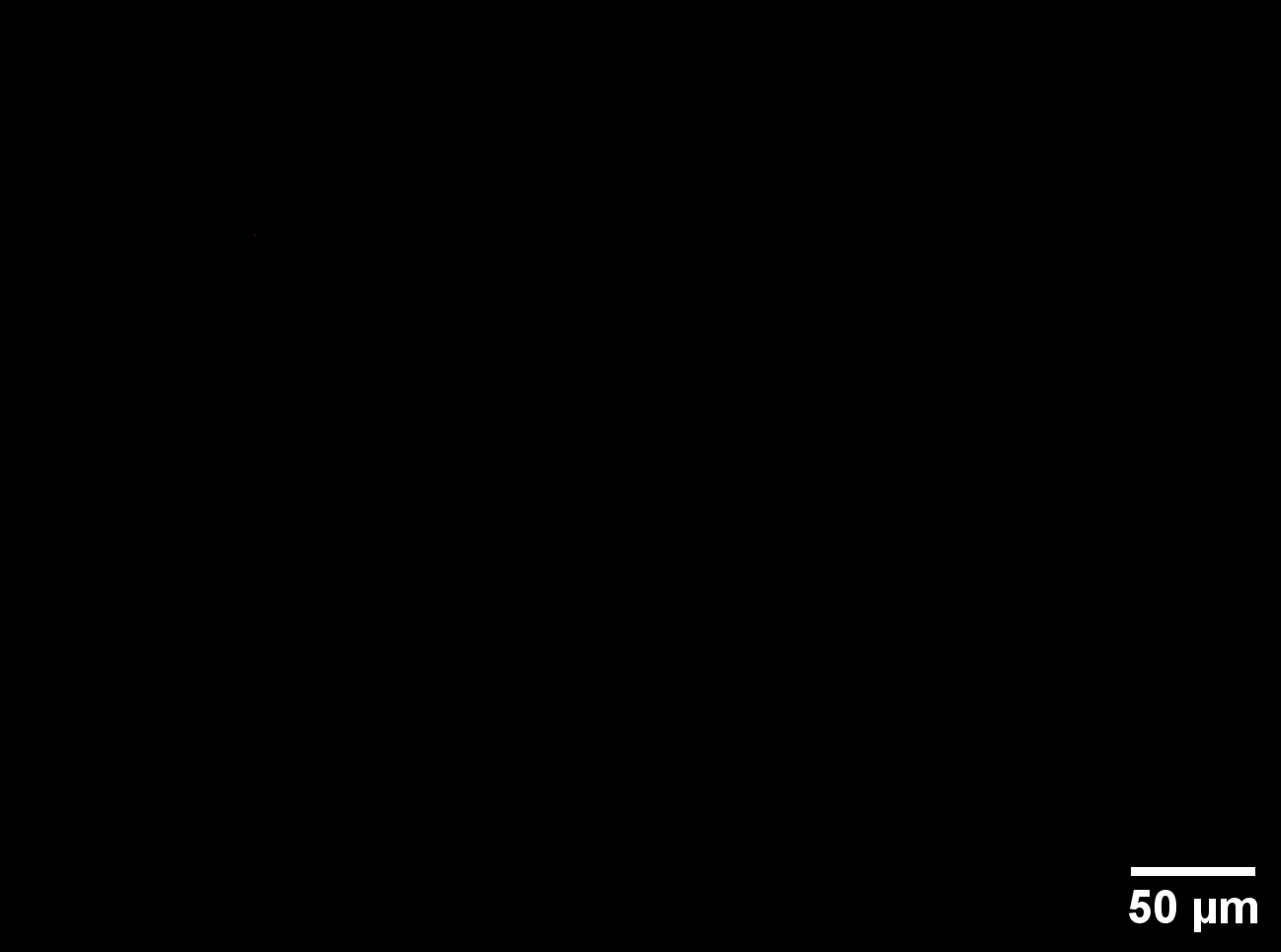

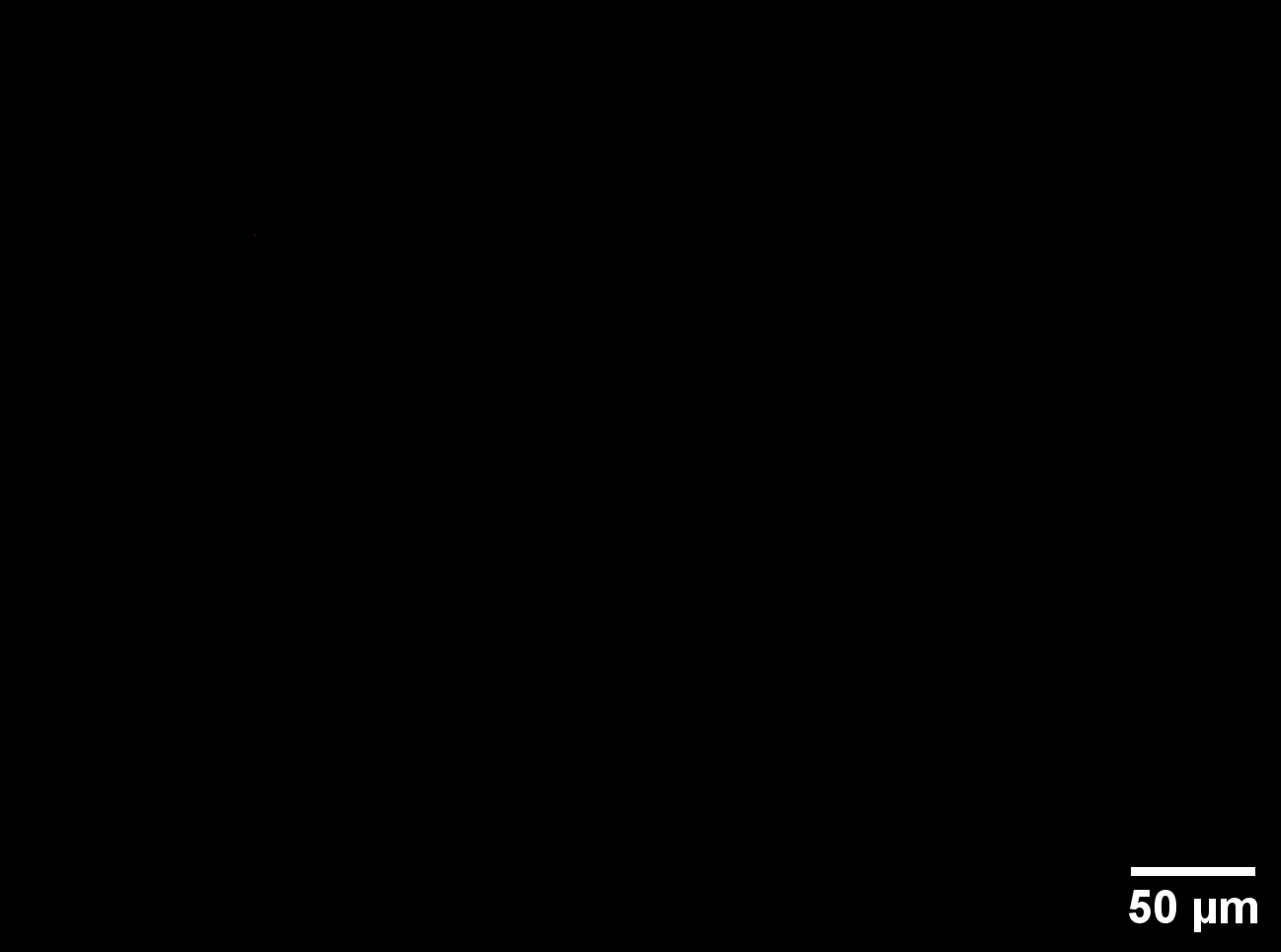


**GFP**

**SiO_2_**

**AgNPs / SiO_2_**

**PI**

**Figure S1.** Epifluorescence microscopic observation of *P. aeruginosa* PAO1-Tn*7-gfp* adhered on SiO_2_ samples and AgNPs-based biomaterials after 90 min of contact under static conditions in WIP. Green (GFP) and red (PI) refer to live and damaged/dead bacteria, respectively.

**S2: Monitoring the number of *P. aeruginosa* PAO1-Tn*7-gfp* counted on the SiO_2_ samples over contact time in WIP in the area of observation under static conditions**

**Figure S2.** Variation in the number of live and dead *P. aeruginosa* PAO1-Tn*7-gfp* cells on SiO_2_ samples over contact time in WIP under static conditions. Results are expressed as cells number ± SD from three independent experiments.

**S3: Adhesion strength, detachment and viability of *P. aeruginosa* PAO1-Tn*7*-*gfp* in contact with AgNPs-based biomaterials after 90 min of contact in WIP under static conditions and under dynamic conditions, following the application of the lowest wall shear stress (0.01 Pa)**

**90 min**


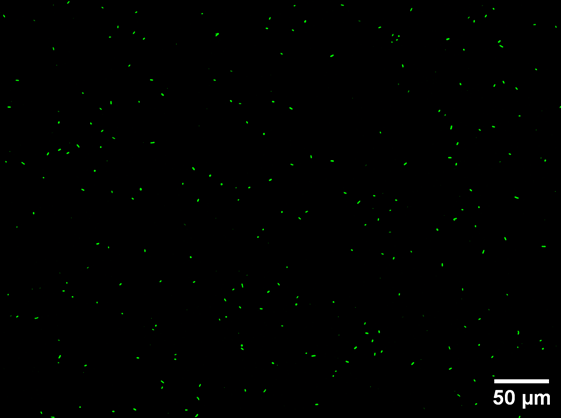

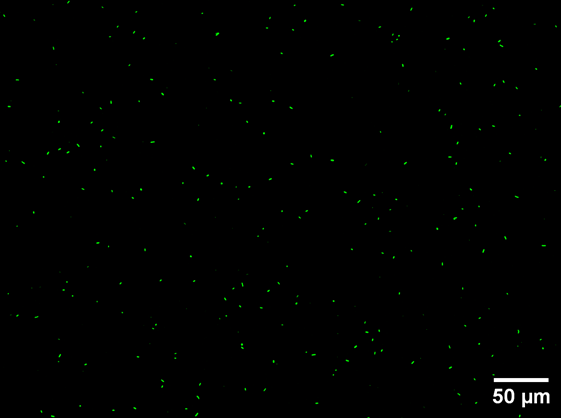


**0.01 Pa**

**(c)**

**(a)**

**SiO_2_**

**
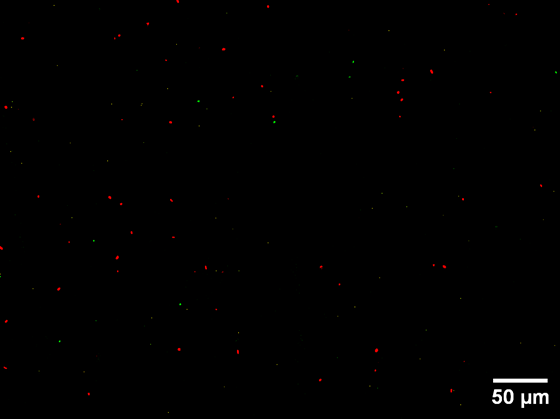

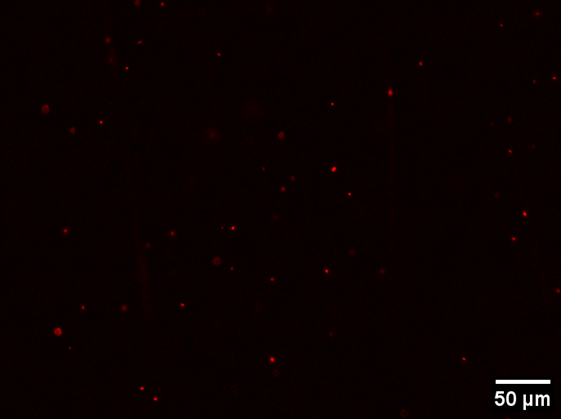
**

**(d)**

**(b)**

**AgNPs / SiO_2_**

**Figure S3.** Epifluorescence microscopic images showing the viability of *P. aeruginosa* PAO1-Tn*7-gfp* on SiO_2_ samples and AgNPs-based biomaterials after 90 min of contact in WIP under static conditions (a,b) and under dynamic conditions (c,d), following the application of the lowest wall shear stress (0.01 Pa). Green (GFP) and red (IP) refer to live and damaged/dead bacteria, respectively.

**S4: Biofilm formation of *P. aeruginosa* PAO1-Tn*7*-*gfp* on SiO_2_ samples under dynamic conditions and resilience of the 72h-old biofilms**


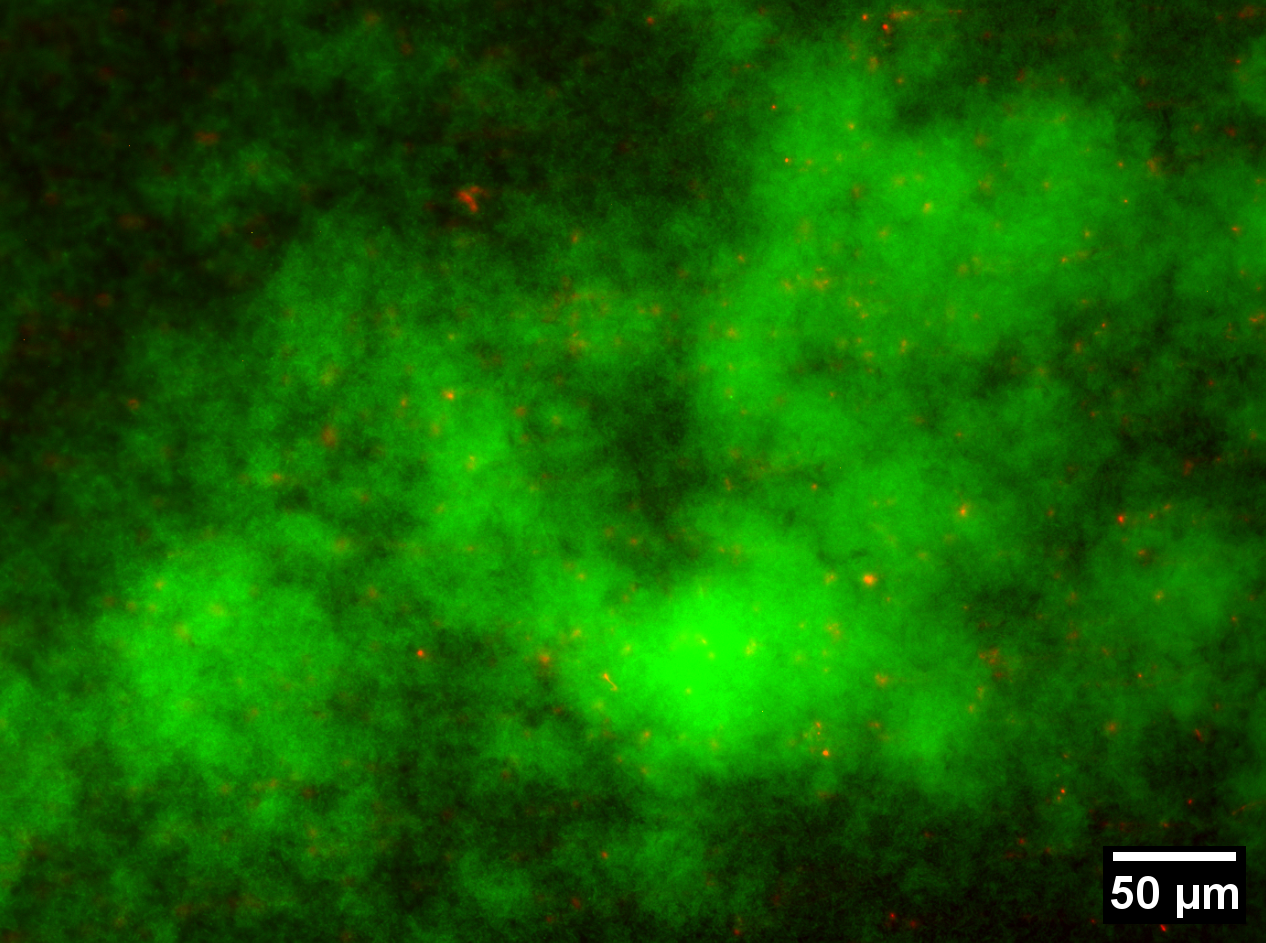

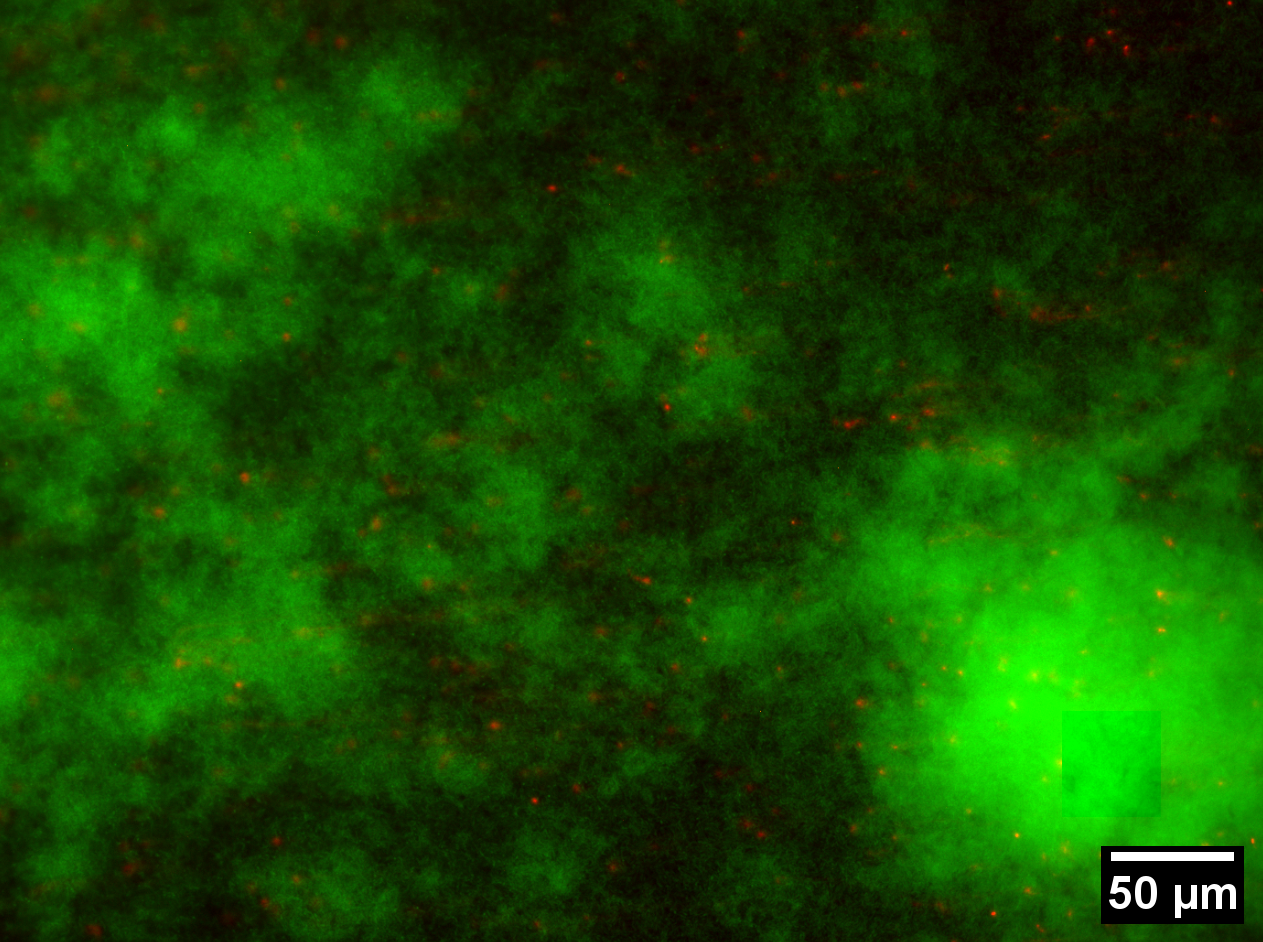


**Figure S4.** *P. aeruginosa* PAO1-Tn*7-gfp* 72h-old biofilms remaining on the SiO_2_ samples (other observation zones) following the application of the highest wall shear stress (16.7 Pa).
